# A Rare Case of Rhabdoid Pancreatic Carcinoma: Prolonged Disease-Free Survival Following Upfront Resection and Adjuvant Chemotherapy

**DOI:** 10.7759/cureus.50145

**Published:** 2023-12-07

**Authors:** Gabriel Land, Benjamin Van Haeringen, Caroline Cooper, Vladimir Andelkovic, Thomas O'Rourke

**Affiliations:** 1 General Surgery, Princess Alexandra Hospital, Brisbane, AUS; 2 Anatomical Pathology, Princess Alexandra Hospital, Brisbane, AUS; 3 Medical Oncology, Princess Alexandra Hospital, Brisbane, AUS; 4 Hepatobiliary Surgery, Princess Alexandra Hospital, Brisbane, AUS

**Keywords:** pancreas, baf47, ini1, switch/sucrose non-fermentable, sarcomatoid, rhabdoid, swi/snf, smarcb1, undifferentiated pancreatic cancer, pancreatic cancer

## Abstract

The rhabdoid subtype of undifferentiated pancreatic carcinoma is rarely reported. The clinical course of this disease is therefore poorly understood, although it is apparently an aggressive malignancy. We herein discuss the case of a 69-year-old man presenting with a rapidly enlarging mass of the pancreatic body and tail who was diagnosed with locally advanced SMARCB1-deficient undifferentiated pancreatic carcinoma with rhabdoid features, treated with radical resection and adjuvant chemotherapy, and has achieved 18-month disease-free survival ongoing at the time of article publication. We identify and contrast our case with 15 similar tumors reported in the English literature, briefly discuss the biology of this tumor, its relationship to malignant rhabdoid tumors of childhood, the role of SMARCB1 and its parent complex switch/sucrose-non-fermentable chromatin remodeling complex (SWI/SNF) in modulating the behavior of pancreatic malignancy, and the potential therapeutic avenues available for SWI/SNF-mutated malignancies.

## Introduction

Pancreatic cancer is a leading contributor to death in Australia, ranking 11th for years of life lost overall and fourth for years of life lost due to cancer deaths [1]. Five-year overall survival is estimated to be as low as 3-8% [2]. Undifferentiated carcinoma (UDC) of the pancreas is an uncommon subtype of pancreatic ductal adenocarcinoma (PDAC) comprising 2-7% of all pancreatic cancers with poorer prognosis compared to conventional PDAC [3-5].

Sarcomatoid UDC is a recognized morphologic pattern of UDC, defined by at least 80% of neoplasm displaying spindle cell features, with or without heterologous differentiation [6]. This pattern includes very rarely reported cases with rhabdoid cells, which share some histological and genetic similarities with other malignancies such as malignant rhabdoid tumors of childhood. These tumors are typified by cells with eccentrically located polygonal nuclei, highly eosinophilic cytoplasm with intracytoplasmic inclusions, a degree of discohesion, and scant myxoid stroma. We have identified in the English literature 15 cases of rhabdoid pancreatic UDC demonstrating mutation or immunohistochemical loss of SMARCB1, a potent tumor suppressor and core subunit of the switch/sucrose-non-fermentable chromatin remodeling complex (SWI/SNF) [7-17]. SWI/SNF is a master regulator of nucleosome occupancy at transcription start sites and thereby of the transcriptome [18]. Mutation, transcriptional repression, or deletion of SMARCB1 is commonly seen in malignant rhabdoid tumors of childhood, where the loss of SMARCB1 function has broad effects on gene transcription and promotes cell-cycle progression and proliferation [18-25]. In humans, SWI/SNF complexes are composed of one of two interchangeable ATPase subunits (BRM/SMARCA2 or BRG1/SMARCA4), an AT-rich DNA-interacting domain (BAF250a/ARID1A or BAF250b/ARID1B), and a variable array of core subunits involved in protein-protein interactions including histone remodeling and interactions with nuclear receptors and transcription factors [24,26,27]. SMARCB1, which encodes Snf5/INI-1/BAF47, is of this latter type.

Loss of SMARCB1, and of other SWI/SNF subunits, is frequently seen in various forms of adult sarcoma and carcinoma; the prognostic and therapeutic significance of this has been only recently elucidated. In a pathological case series of 19 UDCs of the gastrointestinal tract, nine tumors demonstrated rhabdoid morphology; six of these (67%) showed lost or reduced expression of at least one essential SWI/SNF subunit, and three of these tumors were SMARCB1-deficient [28]. Weak SMARCB1 expression occurs in up to 70% of osteosarcomas irrespective of age and correlates with tumor stage, decreased disease-free survival, and decreased progression-free survival [29]. SMARCB1 loss is frequently seen in a rhabdoid phenotype of epithelioid sarcoma and correlates with poor differentiation and metastatic disease in colorectal carcinoma [30-31].

SWI/SNF alterations are common in lung cancer, although SMARCB1 seems less commonly implicated than the ATPase and DNA-interacting domain subunits. ARID1A downregulation or loss is seen in 5-10% of all lung cancers and is associated with reduced 5-year survival in non-small cell lung cancer (NSCLC) [32-33]. Loss of BRG1 or BRM is seen in 30% of NSCLC cell lines; there is concomitant loss in 10% of NSCLC cases which is associated with poor survival [34]. An aggressive, undifferentiated thoracic malignancy, SMARCA4-UT, is typified by loss of the ATPase BRG1 and cytopathological resemblance to SMARCB1/INI1-deficient rhabdoid tumors [35].

We herein describe a case of UDC of the pancreas in a 69-year-old male with immunohistochemical loss of SMARCB1 expression treated with radical resection and adjuvant chemotherapy, demonstrating 18-month disease-free survival at the time of publication.

## Case presentation

**Figure 1:**
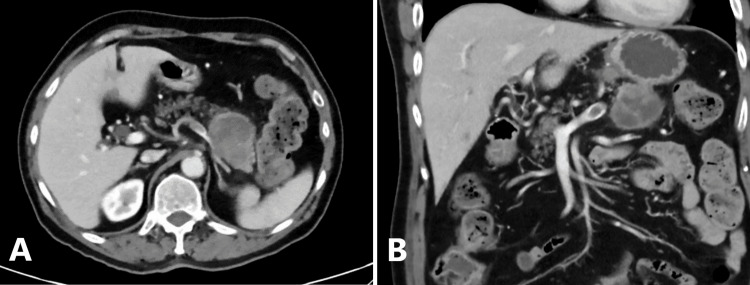
(A) axial and (B) coronal slices of CT at admission, demonstrating 50 x 59 mm (axial) mass of pancreatic body and tail. The tumor encased the splenic artery and was associated with pathological left gastric lymph nodes measuring up to 11 mm.

A 69-year-old man presented to our institution with worsening subacute abdominal pain; serial computed tomography demonstrated a rapidly-growing mass of the pancreatic body and tail (Figure 1) with a maximum radiological dimension of 59 mm, as compared to 43 mm on outpatient imaging four weeks prior. The patient was previously well with no significant medical or surgical history, no known family history of malignancy, and no evidence of exocrine or endocrine pancreatic insufficiency. He was a lifetime non-smoker who consumed alcohol moderately and infrequently. Tumor marker cancer antigen (CA) 19.9 was within the normal range.

**Figure 2:**
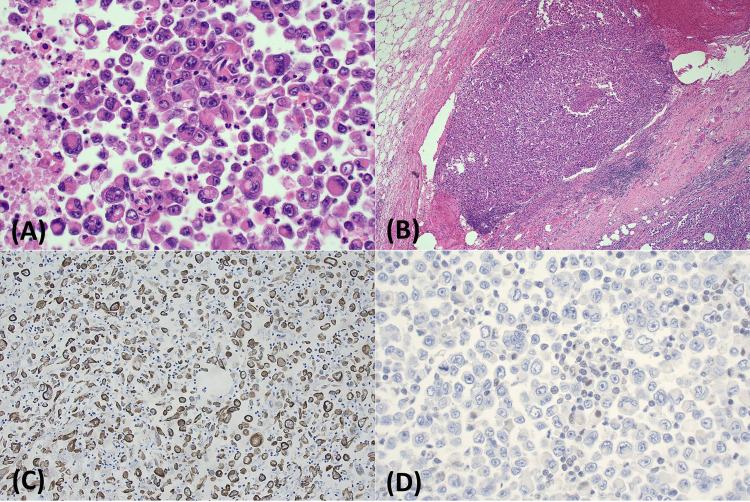
Tumor histopathology. (A) High-power magnification (H&E, 400x) demonstrating sheets of discohesive, pleomorphic, rhabdoid tumor cells including multinucleated forms and necrosis. (B) Low power magnification (H&E, 40x) showing invasion of tumor into a large vein. (C) Tumor cells were strongly positive for MNF116 pan-cytokeratin immunohistochemical stain (200x). (D) Tumor cells showed abnormal loss of nuclear staining for INI1, with retained staining in admixed inflammatory cells (positive internal control, 400x).

Endoscopic ultrasound and core biopsy revealed a malignant neoplasm composed of broad sheets of discohesive epithelioid cells with hyperchromatic, pleomorphic, and eccentrically located nuclei, prominent macronucleoli, and variably prominent rhabdoid morphology. Scattered binucleated and multinucleated cells were noted. No conventional glandular elements were identified. Immunohistochemistry demonstrated strong, diffuse staining for pan-cytokeratin markers (MNF116, AE1/AE3) and focal strong staining for cytokeratin 7, and negative staining for cytokeratin 20, TTF1, CDX2, NKX3.1, ERG, S100 protein, SOX10, and desmin. SMARCB1/INI1 expression was lost, consistent with SMARCB1/INI1-deficient UDC (Figure 2). A subclonal pattern of loss of SMARCA2 was also identified, involving the majority of tumor cells, while SMARCA4 expression was retained. PD-L1 immunohistochemistry demonstrated a combined positive score (CPS) of 90.

Despite rapid interval growth, the tumor remained technically resectable, with no evidence of invasion of the superior mesenteric vascular pedicle. Distal pancreatectomy and splenectomy were performed. Due to extensive desmoplastic change in the retroperitoneum, partial left adrenalectomy was also performed in order to obtain adequate macroscopic posterior margins. Histopathology demonstrated a 66-mm firm, infiltrative mass with central necrosis involving the pancreatic body and tail. It invaded the splenic vein but not the splenic artery and metastatic disease was identified in four of 15 regional lymph nodes. The tumor margin was 7 mm clear from the adrenal gland, though the anterior pancreatic margin was focally involved. The tumor cells showed similar features to those seen at biopsy and were associated with extensive necrosis, numerous mitoses including atypical forms, lymphovascular invasion, and regional lymph node involvement. Some nodal metastases showed conventional acinar cytoarchitectural features which were not identified elsewhere in the tumor. Low-grade pancreatic intraepithelial neoplasia was present in the background ducts, in addition to changes in chronic pancreatitis. Whole exome sequencing revealed loss of heterozygosity of the SMARCB1 locus. The postoperative course was uncomplicated and the patient has completed adjuvant gemcitabine/nab-paclitaxel chemotherapy with no radiological evidence of disease recurrence at 18-month follow-up.

## Discussion

In the largest case series, we identified rhabdoid pancreatic UDC, and median survival was four months [7]. Other series have also supported a poorer one-year overall survival for all forms of pancreatic UDC compared to conventional PDAC, although five-year overall survival is similar [5,36]. Sarcomatoid and anaplastic pancreatic UDC should be distinguished from pancreatic UDC with osteoclast-like giant cells (UDC-OGCs), which may be associated with five-year overall survival exceeding that of conventional PDAC [37-39].

We found no analyses of treatment outcomes in SMARCB1-deficient rhabdoid pancreatic UDC adequately powered to make subtype-specific management recommendations. We searched EMBase, MEDLINE, PubMed, and Cochrane databases, identifying four case series [7,8,13,16] and six case reports [9-12,14,15] which yielded 15 individual cases of SMARCB1-deficient rhabdoid pancreatic UDC (Table 1). Of these, both treatment methods and clinical outcomes were specified for nine cases; three received surgery only [7,9], four received both surgery and chemotherapy [11,12,14,15], one received palliative chemotherapy only [10], and one was palliated without treatment [8]. Most patients died within one year of diagnosis. One patient managed with distal pancreatectomy and adjuvant gemcitabine/paclitaxel had survived 20 months disease-free at the time of publication [14]. Another patient [11] received a Whipple procedure and adjuvant gemcitabine/capecitabine which afforded at least nine months of disease-free survival prior to publication. In our patient’s case, despite locoregionally advanced disease, upfront resection and adjuvant gemcitabine/nab-paclitaxel have thus far yielded 18-month disease-free survival.

**Table 1:** Cases of rhabdoid pancreatic UDC demonstrating immunohistochemical loss or mutation of SMARCB1. UDC: undifferentiated carcinoma

Reference	Patient	Staging	Histology	Immunohistochemistry	Genomic analysis	Treatment	Outcome
Agaimy et al. [7]	76-year-old male (case 11)	NS	Rhabdoid; monomorphic anaplastic	SMARCB1 loss	KRAS wild-type	Surgery	Deceased 1-month post-op
Agaimy et al. [7]	44-year-old female (case 12)	NS	Rhabdoid; monomorphic anaplastic; angiosarcoma-like	SMARCB1 loss	KRAS pGly12Asp amplification	Surgery	NS
Agaimy et al. [7]	72-year-old male (case 13)	NS	Rhabdoid; monomorphic small-cell; pseudopapillary	SMARCB1 loss	KRAS wild-type	Surgery	Deceased 1-week postoperatively
Agaimy et al. [7]	61-year-old male (case 14)	NS	Rhabdoid; monomorphic anaplastic	SMARCB1 loss	KRAS wild-type	Surgery	NS
Sano et al. [8]	68-year-old female	4	Rhabdoid	SMARCB1 loss	NS	Palliative	Deceased 2-weeks post-diagnosis
Hua et al. [9]	59-year-old female	4	Rhabdoid; pseudopapillary-like	SMARCB1 loss; PD-L1 deficient	KRAS wild-type; SMARCB1 deletion	Debulking surgery	Deceased 3-months post-op
Ohike et al. [10]	35-year-old female	4	Rhabdoid; pleomorphic epithelioid	SMARCB1 loss	KRAS wild-type; SMARCB1 wild-type	Palliative TS-1	Deceased 7-months post-diagnosis
Mugaanyi et al. [11]	24-year-old male	3	Rhabdoid; pleomorphic myxoid	SMARCB1 loss	KRAS wild-type; SMARCB1 deletion	Whipple, R0 resection; adjuvant gemcitabine/capecitabine	Disease-free 9-month postoperatively
Tahara et al. [12]	67-year-old female	4	Rhabdoid; monomorphic	SMARCB1 loss	KRAS wild-type	Palliative debulking surgery; palliative chemotherapy	Deceased 2-week postoperatively
Lehrke et al. [13]	Pathology case series	NS	Rhabdoid	SMARCB1 loss; PD-L1-deficient	NS	NS	NS
King et al. [14]	59-year-old female	2b	Rhabdoid; monomorphic epithelioid	SMARCB1 loss; PD-L1 CPS 20	KRAS G12D	Neoadjuvant FOLFIRINOX; distal pancreatectomy; adjuvant gemcitabine/paclitaxel	Disease-free 20-month post-diagnosis
Cho et al. [15]	65-year-old female	4	Rhabdoid; mucinous	NS	SMARCB1 missense	Radical excision; adjuvant radiotherapy	Deceased 1-year postoperatively
Yamamoto et al. [16]	Pathology case series – Case #5	NS	Rhabdoid; anaplastic	SMARCC2 loss	ARID1A missense; SMARCA4 missense; SMARCB1 missense; SMARCC2 missense	NS	NS
Yamamoto et al. [16]	Pathology case series – Case #15	NS	Rhabdoid; anaplastic	SMARCB1 loss; SMARCC1 loss	SMARCA4 missense; SMARCC2 missense	NS	NS
Yamamoto et al. [16]	Pathology case series – Case #3 or 9 or 12 or 14	NS	Rhabdoid; anaplastic; with osteoclast-like giant cells	ARID1A loss; SMARCB1 loss; SMARCC2 loss	ARID1A missense; SMARCA4 missense; SMARCC2 missense	NS	NS

The precise underlying genetic aberrations and potential therapeutic targets in rhabdoid pancreatic UDC remain unclear. Agaimy et al. proposed the existence of two molecularly and morphologically distinct subtypes of rhabdoid pancreatic UDC: an anaplastic monomorphic subtype characterized by SMARCB1 loss without KRAS alteration, and a pleomorphic giant cell subtype characterized by KRAS alterations with intact SMARCB1 expression [7]. However, our patient’s tumor demonstrated significant cellular pleomorphism and giant cells in areas of SMARCB1 loss. Similarly, two previous reports each detailed a rhabdoid pancreatic UDC with concomitant KRAS alteration and loss of SMARCB1 expression, one of which displayed pleomorphic histology with osteoclast-like giant cells [14,16]. A limiting factor of the hypothesis presented by Agaimy et al. is the absence of either immunohistochemical or genomic analysis of SWI/SNF subunits other than SMARCB1. One pathology case series described five rhabdoid pancreatic UDCs (four anaplastic, one sarcomatoid) with mutation or immunohistochemical loss of one or more SWI/SNF subunits [16]; SMARCB1-loss was seen in only two of the anaplastic tumors, and neither with SMARCB1-loss as the sole SWI/SNF aberration. One rhabdoid tumor with intact SMARCB1-expression harbored a missense mutation in the *SMARCB1* gene, with concomitant SMARCC2-loss and mutation of ARID1A and *SMARCA4* genes.

Numerous therapies are emerging to target specific pathways affected by SWI/SNF loss, which may have utility in rhabdoid pancreatic UDC and other malignancies. For both SMARCB1 and ARID1A-mutated tumors, experimental evidence in vitro and murine models suggests that synthetic lethality can be achieved by inhibiting EZH2 methyltransferase of the polycomb regressive complex 2 (PRC2), which is upregulated by loss of SWI/SNF function [40-42]. Tumor regression in a case of SMARCB1-deficient lung cancer has been reported with the use of an inhibitor of aurora kinase A (AurKA), which cooperates with SWI/SNF in mediating topoisomerase II interaction with DNA [43-44]. AurKA inhibition has also been demonstrated to produce synthetic lethality in SMARCA4-mutated murine models of NSCLC [45]. However, human trials have been limited due to relatively low efficacy at tolerable drug doses, particularly with alisertib [46-48]. A novel AurKA inhibitor TAS-119 showed an improved safety profile in phase 1 trials, though phase 2 data are as yet unpublished [49].

The intracytoplasmic aggregates characteristic of rhabdoid tumors have been shown to sequester KEAP1, a ubiquitin ligase adaptor protein, which results in a reduction of NRF2 proteolysis [8]. NRF2 directly upregulates multi-resistance protein 1 (MRP1) expression and contributes to chemoresistance in PDAC and UDC cell lines, suggesting a further axis of potential therapeutic targets [50-52].

Of great clinical interest is the role of immune checkpoint inhibitors in SWI/SNF-mutated pancreatic cancer. Botta et al. reported a case of SMARCB1-deficient pancreatic cancer with a complete response to pembrolizumab and 15-month progression-free survival, although the presence of rhabdoid histology was not specified [53]. The tumor was MMR-deficient, which presents a likely source of immunogenicity; however, Lehrke et al. have presented a series of undifferentiated pancreatic carcinomas in which 15 of 24 (63%) showed PD-L1 enrichment, including four rhabdoid tumors with intact MMR expression [13]. Similarly, Leruste et al. have demonstrated in a murine model of malignant rhabdoid tumor, with knockdown of SMARCB1 as the sole genetic lesion, that infiltration with CD8+ T cell populations enriched with PD-1 expression is characteristic of rhabdoid tumors [54]. They propose disruption of the epigenome secondary to SMARCB1 loss as a prime mediator of immunogenicity.

A series of 4591 solid tumors evaluated with NGS for SWI/SNF mutations compared treatment outcomes with immunotherapy [55]. There was a strong correlation between SWI/SNF mutations and high tumor mutational burden. Of 1001 patients treated with immunotherapy, SWI/SNF mutants had higher rates of disease response, longer progression-free survival, and longer overall survival compared to SWI/SNF wild-type tumors; most of this effect was explained by high tumor mutational burden (TMB) in the SWI/SNF-mutant group. Notably, neither SWI/SNF-mutant pancreatic tumors nor SMARCB1-mutant tumors of any type were prevalent enough to perform meaningful subgroup analysis, and microsatellite instability was not controlled for in the analysis of treatment outcomes according to SWI/SNF status despite the demonstration of higher microsatellite instability rates in the SWI/SNF-mutant cohort. For NSCLC, where perhaps most work has been done to investigate the effectiveness of immunotherapy in SWI/SNF-mutated cancers, results have been inconsistent and prospective data lacking despite promising findings from retrospective reports [56]. Nonetheless, a recent meta-analysis incorporating data from 3416 patients with NSCLC receiving immunotherapy did demonstrate improved progression-free survival in ARID1B-mutant cancers (22.4 vs 4 months) and overall survival in ARID2-mutant cancers (36 vs 11 months) compared to wild-type tumors [57]. When accounting only for tumors with high TMB, all SWI/SNF-mutant NSCLCs demonstrated improved progression-free survival compared to wild-type tumors.

## Conclusions

In summary, rhabdoid histomorphology is rarely seen in cases of UDC of the pancreas; however, when identified, it is often associated with alterations in multiple SWI/SNF subunits, aggressive tumor behavior, and poor clinical outcomes. There is promising pre-clinical and clinical evidence to support the selective use of immune checkpoint inhibitors in non-pancreatic SWI/SNF-mutated tumors, though it is unclear whether the same holds true for SWI/SNF-mutant pancreatic malignancies. Our experience would support upfront resection followed by adjuvant chemotherapy for technically resectable disease in appropriately selected patients with rhabdoid undifferentiated pancreatic carcinoma, but robust data to guide management are lacking given the rarity of this tumor.
